# DW-MRI in assessment of the hypoxic fraction, interstitial fluid pressure, and metastatic propensity of melanoma xenografts

**DOI:** 10.1186/1471-2407-14-92

**Published:** 2014-02-15

**Authors:** Tord Hompland, Christine Ellingsen, Kanthi Galappathi, Einar K Rofstad

**Affiliations:** 1 Group of Radiation Biology and Tumor Physiology, Department of Radiation Biology, Institute for Cancer Research, Oslo University Hospital, Nydalen, Box 4953, Oslo N-0424, Norway

**Keywords:** DW-MRI, Hypoxia, Interstitial fluid pressure, Metastasis, Cell density

## Abstract

**Background:**

Cancer patients with primary tumors showing extensive hypoxia and highly elevated interstitial fluid pressure (IFP) have poor prognosis. The potential of diffusion-weighted magnetic resonance imaging (DW-MRI) in assessing the hypoxic fraction, IFP, and metastatic propensity of tumors was investigated in this study.

**Methods:**

A-07 and R-18 melanoma xenografts were used as general models of human cancer. DW-MRI was performed at 1.5 T, and maps of the apparent diffusion coefficient (ADC) were produced with in-house-made software developed in Matlab. Pimonidazole was used as a hypoxia marker. Tumor cell density and hypoxic fraction were assessed by quantitative analysis of histological sections. IFP was measured with a Millar catheter. Metastatic propensity was determined by examining tumor-bearing mice for pulmonary micrometastases post mortem.

**Results:**

ADC decreased with increasing tumor cell density, independent of whether the A-07 and R-18 data were analyzed separately or together. In the A-07 line, ADC decreased with increasing hypoxic fraction and increasing IFP and was lower in metastatic than in nonmetastatic tumors, and in the R-18 line, ADC decreased with increasing hypoxic fraction. There was a strong inverse correlation between ADC and hypoxic fraction as well as between ADC and IFP across the two tumor lines, primarily because low ADC as well as high hypoxic fraction and high IFP were associated with high cell density.

**Conclusion:**

Low ADC is a potentially useful biomarker of poor prognosis in cancer, since low ADC is mainly a consequence of high cell density, and high cell density may lead to increased hypoxia and interstitial hypertension and, therefore, increased microenvironment-associated metastasis.

## Background

Diffusion-weighted magnetic resonance imaging (DW-MRI) is an imaging technique that is sensitive to the Brownian motion of water in tissues [1,2]. Quantitative information can be obtained, and this information is usually displayed as maps of a parameter called the apparent diffusion coefficient (ADC). A high ADC indicates relatively free Brownian motion of water, whereas a low ADC is indicative of restricted motion [2]. The Brownian motion of water is restricted by organelles, cell membranes, and extracellular fibers, and consequently, ADC maps can provide information on the microstructure and composition of tissues. For example, studies of tumors have shown that high cell density gives rise to low ADC values, whereas high ADC values are measured in necrotic tissue [1,2].

DW-MRI has several potentially useful applications in clinical oncology and is emerging as a powerful tool in the management of cancer patients. This tool is currently being used to distinguish malignant from benign lesions, to differentiate post-therapeutic changes from residual tumor tissue, to detect recurrent tumors, lymph node involvement, and distant metastases, and to predict and monitor response to treatment [3-5]. DW-MRI appears to be particularly promising for detecting metastatic disease, both in regional lymph nodes and distant organs [3]. Furthermore, several studies have suggested that ADC may increase rapidly during and after radiation therapy in radiosensitive tumors, an effect that has been attributed to increased water diffusion due to cell death, development of edema, and increased microvessel leakiness [4,5]. However, the prognostic value of the pretreatment ADC map and its potential as a biomarker of outcome of treatment are unclear. On the one hand, low pretreatment ADC values have been shown to correlate with several established histological and clinical biomarkers of poor prognosis [6,7]. On the other hand, several studies have suggested that high pretreatment ADC values are indicative of poor response to treatment, possibly because of an association between tumor hypoxia and necrosis [8,9].

Detailed studies of associations between pretreatment ADC values and the microenvironment of tumors are needed to evaluate the usefulness of DW-MRI as a tool for providing biomarkers of tumor aggressiveness and treatment outcome. Several parameters of the tumor microenvironment including the level of hypoxia and interstitial hypertension have been shown to be associated with metastatic growth, treatment failure, and poor disease-free and overall survival rates [10-12]. In the study reported here, human melanoma xenografts were used to investigate whether DW-MRI may provide information on these microenvironmental parameters and the microenvironment-associated metastatic propensity of tumors. The A-07 and R-18 melanoma lines were selected for the study because the tumors of these lines differ substantially in cell density and do not show necrotic regions [13].

## Methods

### Tumor models

A-07 and R-18 human melanoma xenografts growing in adult female BALB/c *nu*/*nu* mice were used as tumor models [13]. Tumors were initiated from cells cultured in RPMI-1640 (25 mmol/L HEPES and l-glutamine) medium supplemented with 13% bovine calf serum, 250 mg/L penicillin, and 50 mg/L streptomycin. Approximately 3.5 × 10^5^ cells in 10 μL of Hanks’ balanced salt solution were inoculated intradermally in the hind leg. After the cell inoculation, the mice were monitored daily for tumor growth. Experiments were started when the tumors had grown to a volume of ~400 mm^3^. DW-MRI and IFP measurements were carried out with mice anesthetized with fentanyl citrate (0.63 mg/kg), fluanisone (20 mg/kg), and midazolam (10 mg/kg). Animal care and experimental procedures were in concordance with the U.S. Public Health Service Policy on Humane Care and Use of Laboratory Animals as well as Norwegian legislation and were approved by the Norwegian National Animal Research Authority.

### DW-MRI

DW-MRI was carried out with a 1.5-T whole-body clinical scanner (Signa; General Electric, Milwaukee, WI) and a slotted tube resonator transceiver coil constructed for mice. The coil was insulated with styrofoam to prevent excessive heat loss from the animals. The body core temperature of the mice was kept at 37–38°C during imaging by using a thermostatically regulated heating pad. The tumors were positioned in the isocenter of the magnet and were imaged axially in a single section through the tumor center by applying a diffusion-weighted single-shot fast spin echo sequence with ETL = 84 and TR = 5002 ms. Images were recorded at a spatial resolution of 0.39  ×  0.39  ×  2.0 mm^3^ by using an image matrix of 256 × 256, a field of view of 10 × 10 cm, and 10 excitations. Diffusion sensitization gradients were applied in six noncollinear directions with the following x, y, and z physical gradient combinations: [1 0 1], [-1 0 1], [0 1 1], [0 1 -1], [1 1 0], [-1 1 0]. Five different diffusion-weightings with diffusion encoding constants of *b* = 200, 400, 600, 800, and 1000 s/mm^2^ and corresponding echo times of TE = 85.0, 95.5, 103.0, 108.9, and 113.9 ms were used. The diffusion weightings were achieved by using gradient durations of *δ* = 11.6, 16.7, 20.8, 24.3, and 27.5 ms, a gradient interval of Δ = 51 ms, and a gradient strength of *G*_D_ = 21 mT/m. An image without diffusion weighting (*b* = 0) was recorded for each TE value to compensate for the different TE values associated with the different *b* values. The *b* = 0 images were not included in the calculation of ADC. The total scan time was ~27 min.

ADC maps were produced with in-house-made software developed in Matlab. Briefly, the directional diffusion images were averaged on a voxel-by-voxel basis to non-directional diffusion images. ADC values were calculated for each voxel by fitting the mono-exponential model equation to five-point plots of signal intensity (*S*) *versus b*:

$$\log\left(\frac{S\left(b,\text{TE}\right)}{S\left(b=0,\text{TE}\right)}\right)=-b\cdot \text{ADC}+c$$

by using a linear least square fit algorithm. A mono-exponential model and *b* values from 200 to 1000 s/mm^2^ were chosen to avoid confounding effects of perfusion, as recommended by Padhani et al. [14]. Parametric images of ADC and the correlation coefficients of the curve fits were generated with the SigmaPlot software. It was verified that the mono-exponential model gave curve fits with correlation coefficients ≥ 0.98 in more than 95% of the voxels in all tumors. Median ADC, calculated from the ADC values of the individual voxels, was used as a parameter for the ADC of a tumor.

### Cell density

Tumor cell density was determined by stereological analysis of histological sections prepared from tissue fixed in phosphate-buffered 4% paraformaldehyde. The sections were stained with hematoxylin to render cell nuclei clearly visible. The density of cell nuclei was measured by using the method of Brammer and Jung [15], and the density of cells was assumed to be equal to the density of nuclei. Five fields of view, each corresponding to 500 - 600 cells, were analyzed for each tumor. Further details of the procedure have been reported elsewhere [16].

### Hypoxia

Pimonidazole [1-[(2-hydroxy-3-piperidinyl)-propyl]-2-nitroimidazole] was administered intraperitoneally in a dose of 30 mg/kg and used as a marker of tumor hypoxia [17]. The tumors were fixed in phosphate-buffered 4% paraformaldehyde ~4 h after the pimonidazole administration. Histological sections were immunostained for hypoxia by using a peroxidase-based indirect staining method [17]. An anti-pimonidazole rabbit polyclonal antibody (gift from Professor Raleigh, University of North Carolina) was used as primary antibody. Diaminobenzidine was used as chromogen, and hematoxylin was used for counterstaining. Three cross-sections were examined for each tumor. The area of the tumor tissue staining positive for pimonidazole was identified by image analysis [17]. Hypoxic fraction (HF_Pim_) was calculated as pimonidazole-positive area divided by total tissue area.

### Interstitial fluid pressure

IFP was measured in the tumor center with a Millar SPC 320 catheter equipped with a 2 F Mikro-Tip transducer (Millar Instruments, Houston, TX) [18]. The catheter was connected to a computer via a Millar TC-510 control unit and a preamplifier. Data acquisition was carried out by using the LabVIEW software (National Instruments, Austin, TX).

### Pulmonary metastases

Microscopic pulmonary metastases were detected by histological examination. Resected lungs were fixed in phosphate-buffered 4% paraformaldehyde and embedded in paraffin. Histological sections were cut from each lobe at 100-μm intervals and stained with hematoxylin and eosin. Groups of five or more tumor cells were scored as a metastasis.

### Experimental design

Three individual experimental series were carried out one subsequent to the other. In the first series, possible associations between ADC and tumor cell density were investigated. In the second series, we investigated whether HF_Pim_ and/or IFP may differ between tumors with low and tumors with high cell densities. Possible associations between ADC on the one hand and HF_Pim_, IFP, and metastatic propensity on the other were investigated in the third experimental series. The mice were first subjected to DW-MRI, then tumor IFP was measured while the mice were still under anesthesia, and then the mice were euthanized and the tumors and lungs were resected and prepared for assessment of HF_Pim_ and metastatic status, respectively.

### Statistical analysis

Experimental data are presented as mean ± SE unless otherwise stated. Curves were fitted to data by regression analysis. Correlations between variables were searched for by using the Pearson product moment correlation test. Statistical comparisons of data were carried out by using the Student’s *t* test when the data complied with the conditions of normality and equal variance. Under other conditions, comparisons were carried out by non-parametric analysis using the Mann-Whitney rank sum test. The Kolmogorov-Smirnov method was used to test for normality. Probability values (*P*) and correlation coefficients (*R*^2^) were calculated by using the SigmaStat statistical software. A significance criterion of *P* < 0.05 was used.

## Results

Altogether, ADC maps were generated for 35 A-07 and 36 R-18 tumors, IFP and HF_Pim_ were measured in 39 A-07 and 38 R-18 tumors, and cell density was determined for 24 A-07 and 22 R-18 tumors. The A-07 tumors showed a median ADC of 1.0 × 10^-3^ mm^2^/s, a median IFP of 12 mmHg, a median HF_Pim_ of 3.7%, and a median cell density of 1.8 × 10^5^ cells/mm^3^. The corresponding values for the R-18 tumors were 5.3 × 10^-4^ mm^2^/s (ADC), 28 mmHg (IFP), 5.8% (HF_Pim_), and 4.2 × 10^5^ cells/mm^3^ (cell density).

**Figure 1:**
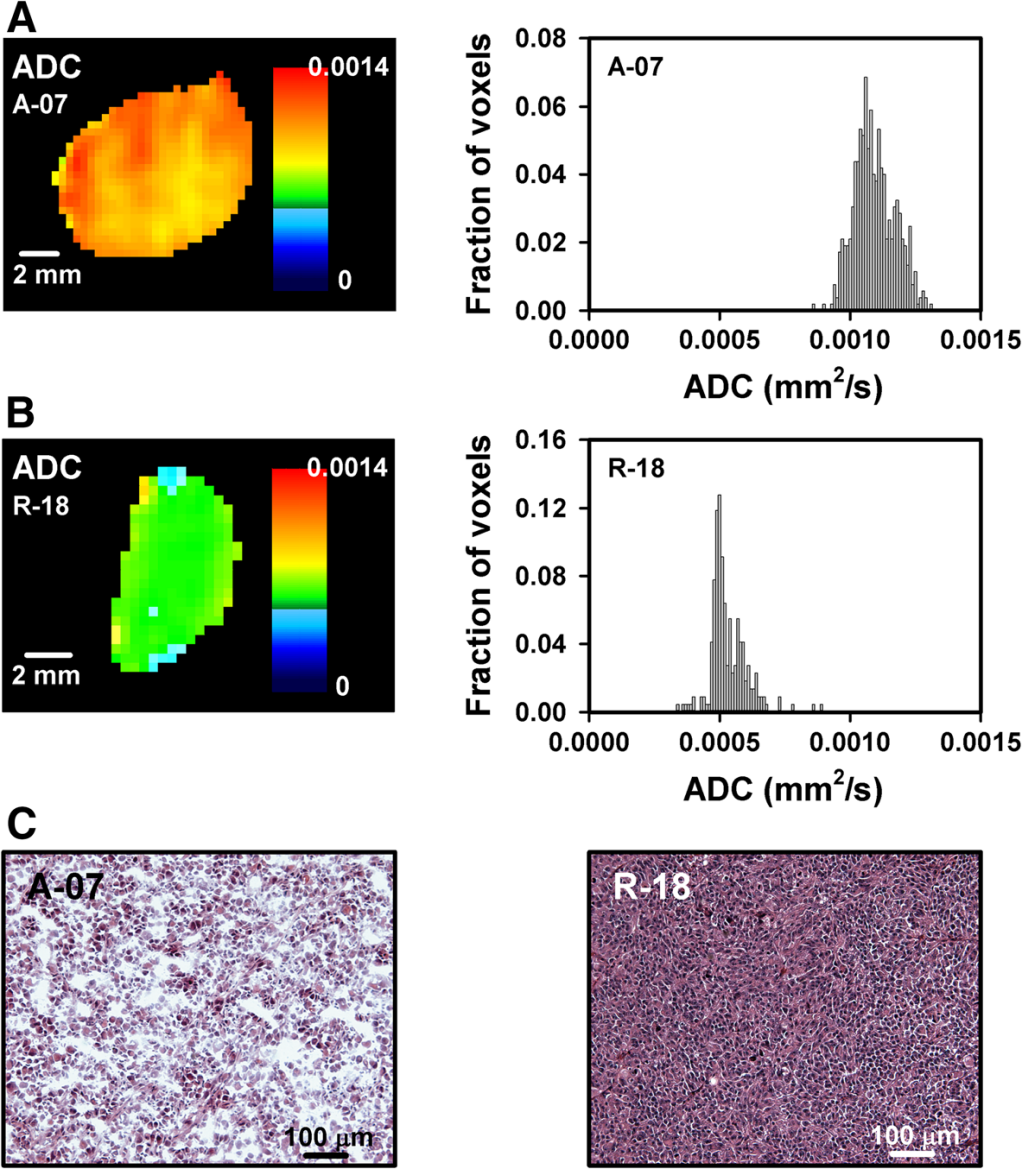
**DW-MRI and histological data showing that A-07 tumors had higher ADC values and lower cell densities than R-18 tumors.** The ADC map and the corresponding ADC frequency distribution of a representative A-07 tumor **(A)** and a representative R-18 tumor **(B)** and the histological appearance of a representative A-07 and a representative R-18 tumor **(C)**.

ADC was considerably higher in the A-07 than in the R-18 tumors. This is exemplified in Figure 1, which shows the ADC map and the corresponding ADC frequency distribution of a representative A-07 tumor (Figure 1A) and a representative R-18 tumor (Figure 1B). The difference in ADC was most likely a consequence of the lower cell density in the A-07 than in the R-18 tumors. Figure 1C shows representative images of the histology of an A-07 (left) and an R-18 (right) tumor, illustrating the difference in cell density.

**Figure 2:**
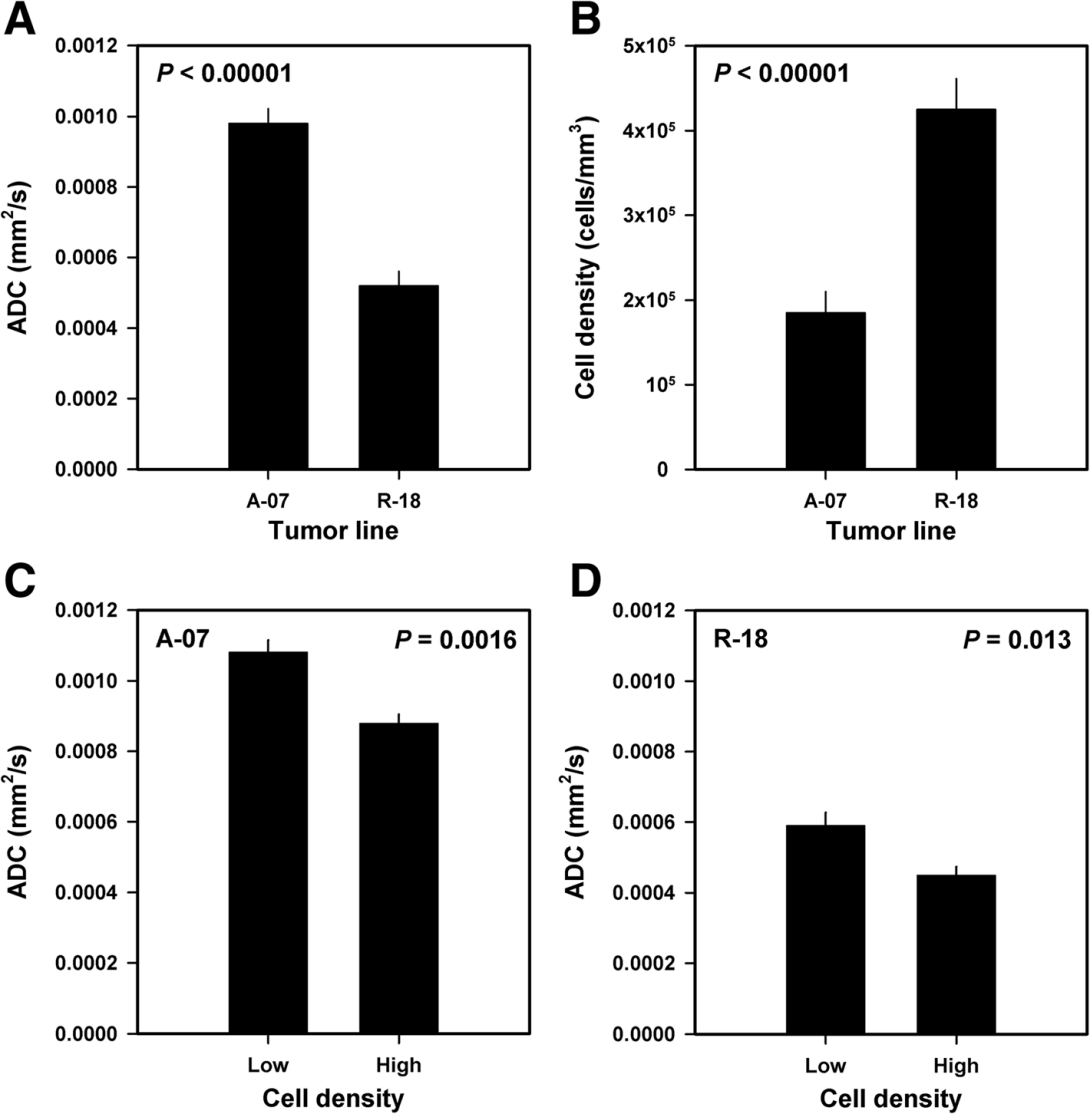
**DW-MRI and histological data for A-07 and R-18 melanoma xenografts showing that high ADC was associated with low cell density.** Median ADC **(A)** and cell density **(B)** for A-07 and R-18 tumors, median ADC for A-07 tumors with low and high cell densities (cell densities below and above the median value of 1.8 × 10^5^ cells/mm^3^) **(C)**, and median ADC for R-18 tumors with low and high cell densities (cell densities below and above the median value of 4.2 × 10^5^ cells/mm^3^) **(D)**. Columns and bars represent mean values and standard errors [n = 10 **(A and B)**, n = 5 **(C and D)**].

Quantitative studies of possible associations between ADC and cell density were carried out in 10 tumors of each line. Compared with the R-18 tumors, the A-07 tumors showed higher ADC values (*P* < 0.00001, Figure 2A) and lower cell densities (*P* < 0.00001, Figure 2B). The tumors with cell densities below the median value had higher ADC values than the tumors with cell densities above the median value [*P* = 0.0016, Figure 2C (A-07); *P* = 0.013, Figure 2D (R-18)]. The inverse relationship between ADC and cell density was statistically significant at the single tumor level, both within each melanoma line [*P* = 0.00086 (A-07); *P* = 0.0023 (R-18)] and across the two melanoma lines (*P* < 0.00001).

**Figure 3:**
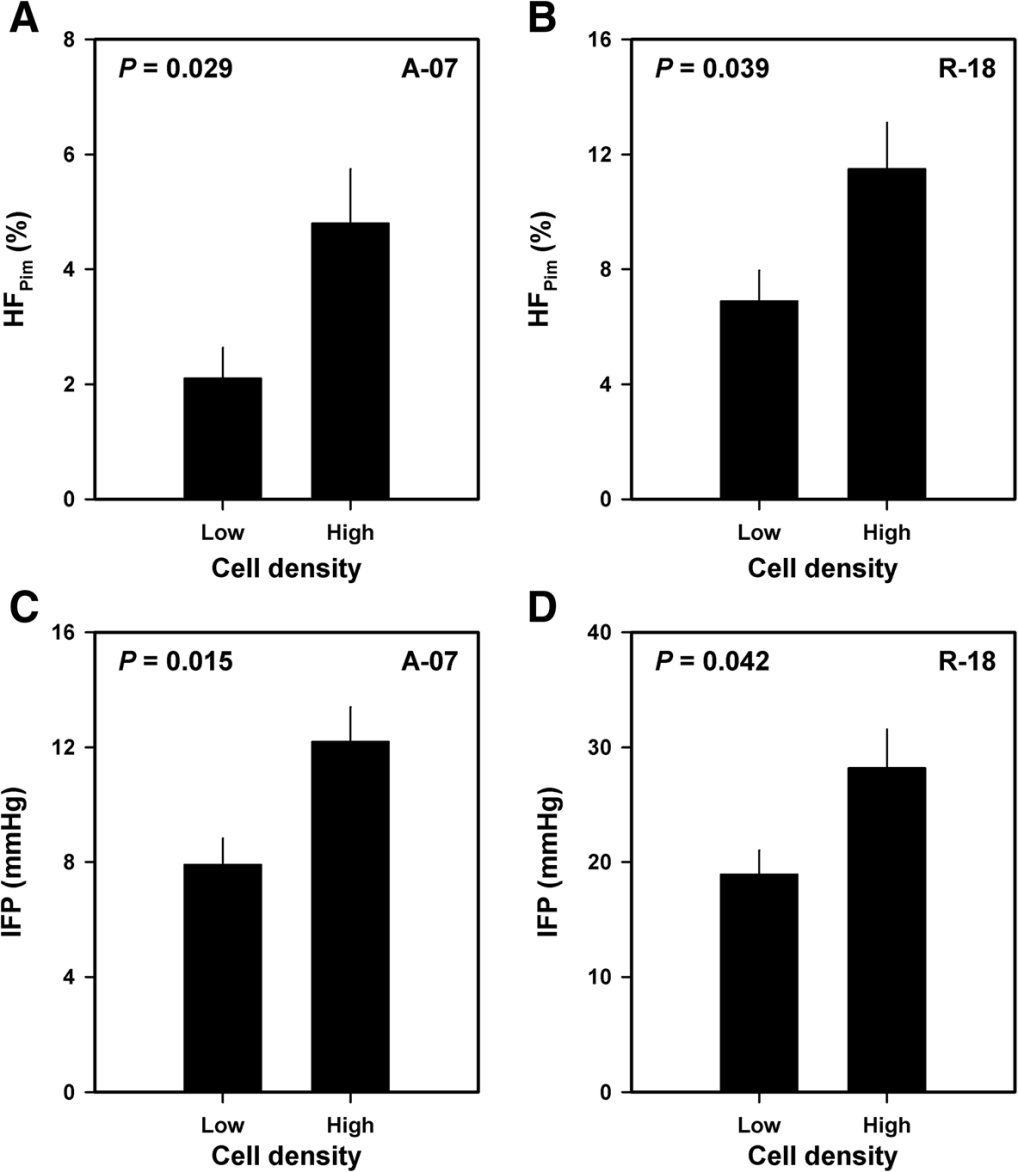
**Hypoxia, IFP, and cell density data for A-07 and R-18 melanoma xenografts showing that high HF**_**Pim **_**and high IFP were associated with high cell density.** HF_Pim_ for A-07 tumors with low and high cell densities (cell densities below and above the median value of 1.8 × 10^5^ cells/mm^3^) **(A)**, HF_Pim_ for R-18 tumors with low and high cell densities (cell densities below and above the median value of 4.2 × 10^5^ cells/mm^3^) **(B)**, IFP for A-07 tumors with low and high cell densities **(C)**, and IFP for R-18 tumors with low and high cell densities **(D)**. Columns and bars represent mean values and standard errors (n = 7 for A-07 and n = 6 for R-18).

Fourteen A-07 and 12 R-18 tumors were subjected to measurement of IFP and subsequent histological examinations to investigate whether HF_Pim_ and/or IFP may differ between tumors with low and tumors with high cell densities. Compared with the tumors with cell densities below the median value, the tumors with cell densities above the median value had both higher HF_Pim_ [*P* = 0.029, Figure 3A (A-07); *P* = 0.039, Figure 3B (R-18)] and higher IFP [*P* = 0.015, Figure 3C (A-07); *P* = 0.042, Figure 3D (R-18)].

**Figure 4:**
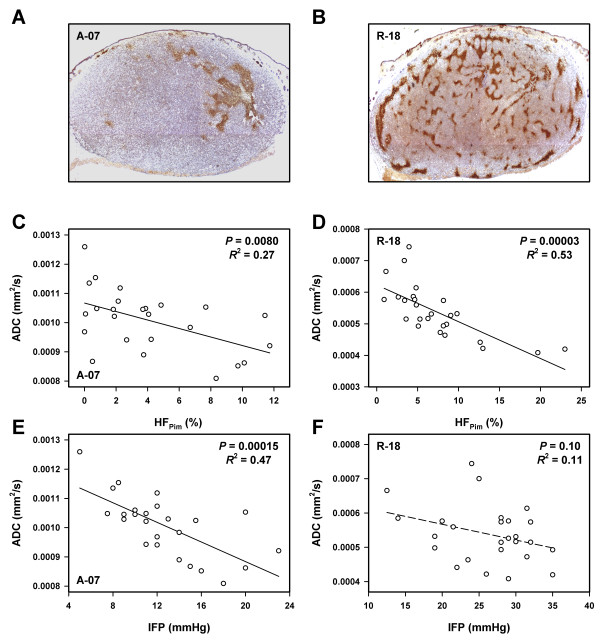
**DW-MRI, hypoxia, and IFP data for A-07 and R-18 melanoma xenografts showing that ADC decreased with increasing HF**_**Pim **_**and increasing IFP.** Histological sections of a representative A-07 tumor **(A)** and a representative R-18 tumor **(B)** stained with anti-pimonidazole antibody to visualize hypoxic tissue, plots of median ADC *versus* HF_Pim_ for A-07 **(C)** and R-18 **(D)** tumors, and plots of median ADC *versus* IFP for A-07 **(E)** and R-18 **(F)** tumors. Points represent single tumors. Curves were fitted to data by regression analysis.

To search for associations between ADC on the one hand and HF_Pim_, IFP, and metastatic propensity on the other, 25 A-07 tumors and 26 R-18 tumors were subjected to DW-MRI and measurement of IFP before the host mice were euthanized and the tumors and lungs were resected and prepared for histological examinations. Histological sections of representative tumors stained for hypoxia are presented in Figure 4A (A-07) and Figure 4B (R-18). HF_Pim_ differed among the individual tumors from 0 to 12% (A-07) and from 1 to 23% (R-18). ADC decreased with increasing HF_Pim_, both in the A-07 (*P* = 0.0080, Figure 4C) and the R-18 (*P* = 0.00003, Figure 4D) tumors. IFP differed among the individual tumors from 5 to 23 mmHg (A-07) and from 12 to 35 mmHg (R-18). ADC decreased significantly with increasing IFP in the A-07 tumors (*P* = 0.00015, Figure 4E), but not in the R-18 tumors (*P* = 0.10, Figure 4F). Furthermore, by analyzing the A-07 and R-18 data together, it was shown that there was an inverse correlation between ADC and HF_Pim_ and between ADC and IFP across the two melanoma lines [*P* = 0.00009 (HF_Pim_); *P* < 0.00001 (IFP)].

**Figure 5:**
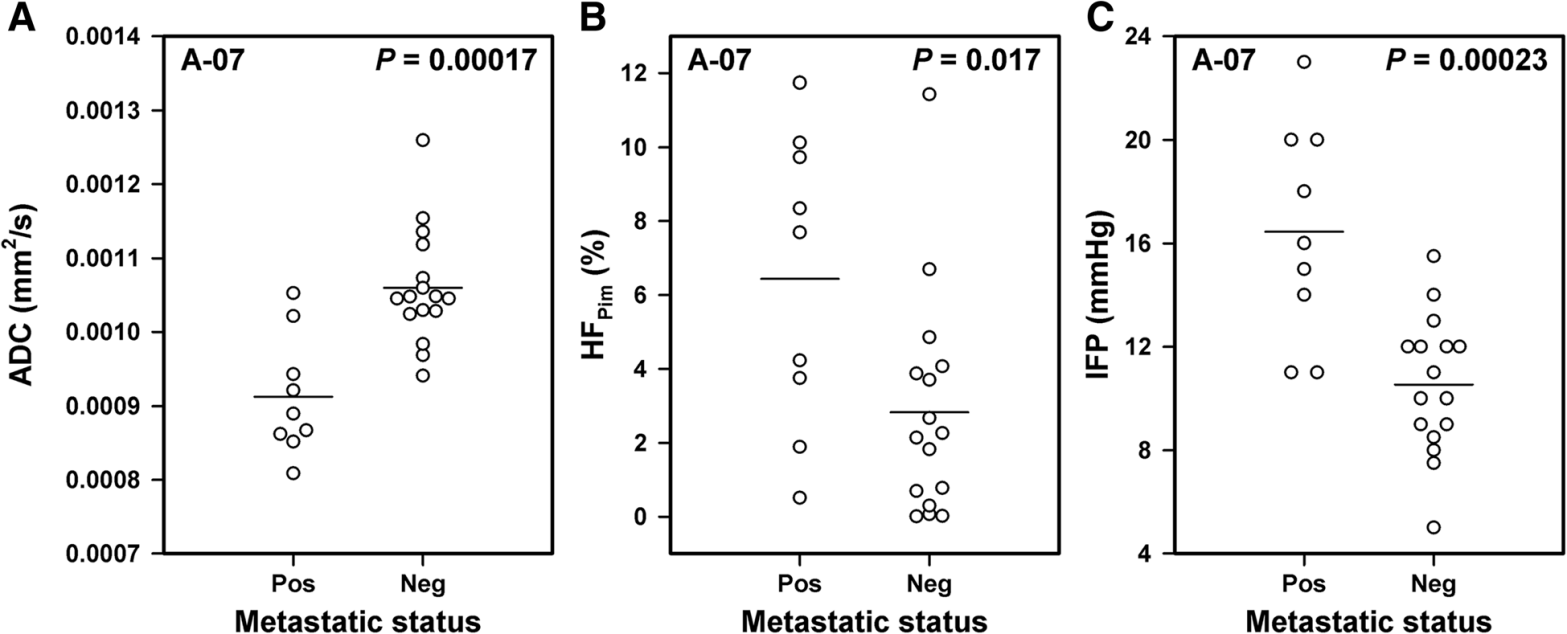
**DW-MRI, hypoxia, IFP, and metastasis data for A-07 melanoma xenografts showing that metastatic tumors had lower ADC, higher HF**_**Pim**_**, and higher IFP than nonmetastatic tumors.** Median ADC **(A)**, HF_Pim_**(B)**, and IFP **(C)** for metastatic and nonmetastatic tumors. Points represent single tumors. Horizontal bars indicate mean values.

Pulmonary micrometastases were detected in 9 of the 25 mice bearing A-07 tumors, whereas the remaining 16 mice were metastasis-negative. Compared with the tumors of the metastasis-negative mice, the tumors of the metastasis-positive mice had lower ADC values (*P* = 0.00017, Figure 5A), higher HF_Pim_ (*P* = 0.017, Figure 5B), and higher IFP (*P* = 0.00023, Figure 5C). Metastatic deposits were not detected in any of the mice bearing R-18 tumors.

## Discussion

The possibility that the pretreatment ADC map may provide a useful biomarker of the outcome of cancer has generated a large number of clinical investigations involving several tumor types [3-8]. Some studies suggest that poor clinical outcome is associated with low ADC values, whereas high ADC values have been found to be associated with poor outcome in other studies [3,5,9,19]. The reasons for these apparently conflicting observations have not been identified, possibly because DW-MRI has not been combined with detailed studies of biological and physiological characteristics of the imaged tissue.

In the present preclinical study, tumors subjected to DW-MRI were examined with respect to cell density, fraction of hypoxic tissue, level of interstitial hypertension, and metastatic status. Tumors with low ADC values were found to have high cell densities, consistent with the general theory that low ADC indicates restricted Brownian motion of water [1-3]. Furthermore, low ADC values in tumors were associated with high HF_Pim_, high IFP, and elevated metastatic propensity in A-07 tumors and with high HF_Pim_ in R-18 tumors.

Tumor hypoxia is a result of an imbalance between the rate of oxygen supply and the rate of oxygen consumption [20]. The rate of oxygen supply is determined primarily by the vascular architecture and the rate of blood flow, whereas the rate of oxygen consumption is determined primarily by the respiratory activity of the tumor cells and the cell density. In the A-07 and R-18 lines, tumors with high cell densities showed higher HF_Pim_ than tumors with low cell densities, suggesting that the intertumor heterogeneity in fraction of hypoxic tissue was influenced significantly by the intertumor heterogeneity in cell density. The inverse correlations between ADC and HF_Pim_ in A-07 and R-18 tumors were therefore most likely an indirect consequence of the relationship between ADC and cell density.

Interstitial hypertension in tumors is primarily a consequence of high resistance to blood flow, low resistance to transcapillary fluid flow, decreased interstitial hydraulic conductivity, and impaired lymphatic drainage [21]. In tumors with highly permeable blood vessels like the A-07 and R-18 tumors, the intertumor heterogeneity in IFP is mainly a result of intertumor heterogeneity in resistance to both blood flow and interstitial fluid flow [22]. High cell density is associated with high resistance to interstitial fluid flow [23], and furthermore, cell division in rapidly growing tumors with high cell densities may cause mechanical pressure on vessel walls, leading to local vessel narrowings and, hence, increased resistance to blood flow [24]. IFP was higher in tumors with high cell densities than in tumors with low cell densities in the A-07 and R-18 lines. The association between ADC and IFP observed here was therefore most likely an indirect consequence of the relationship between ADC and cell density.

There is significant evidence from clinical studies that poor disease-free and overall rates in cancer are associated with extensive hypoxia and interstitial hypertension in the primary tumor [10-12]. Preclinical studies have revealed that pulmonary and lymph node metastasis is promoted by tumor hypoxia [25,26] and highly elevated IFP [27,28]. Previous studies have shown that metastasis is associated with hypoxia and the level of interstitial hypertension also in A-07 tumors [28,29]. These observations were confirmed in the present work, which showed that metastatic A-07 tumors had higher HF_Pim_ and higher IFP than their nonmetastatic counterparts. The lower ADC in the metastatic than in the nonmetastatic A-07 tumors was most likely an indirect consequence of higher cell densities in the metastatic tumors, since both high HF_Pim_ and high IFP were associated with metastasis, high cell density, and low ADC.

Taken together, our data suggest that low rather than high ADC values are indicative of tumor aggressiveness and poor prognosis in cancer. This suggestion is consistent with some clinical investigations, which have shown that low ADC values in untreated tumors may be associated with several established biomarkers of poor prognosis in breast [6], prostate [7], and lung cancer [30]. Recent clinical studies have also provided significant evidence that low ADC values may be a characteristic feature of primary tumors that are likely to metastasize [31]. Moreover, aggressive tumors have been demonstrated to have lower ADC values than indolent tumors in patients with localized prostate cancer [32], and the ADC of the primary tumor has been found to be lower in patients with metastatic disease than in patients without detectable metastases in high-grade urothelial carcinoma of the bladder [33].

In contrast, some preclinical and clinical studies have suggested that poor response to treatment is associated with high pretreatment ADC values [8,9]. High ADC values may be associated with treatment resistance if the treatment resistance is caused by hypoxia and the tumors show significant hypoxia-associated necrosis. Proper evaluation of the prognostic and predictive potential of DW-MRI may require that voxels in necrotic tumor regions are excluded from ADC maps, since ADC maps may be confounded significantly by the highly elevated ADC values seen in necrotic tissue. Necrotic tumor regions may be identified by combining DW-MRI with contrast-enhanced MRI [34]. It is important to note that A-07 and R-18 tumors in general do not develop necrotic regions until they reach a volume of 700 - 800 mm^3^[13], and necrosis was not seen in any of the histological sections examined in the present work.

In preclinical studies, each tumor line represents a single patient, whereas individual tumors of the same line represent copies of the same tumor. These tumor copies have the same genetic background, but may show substantially different physiological microenvironments owing to the stochastic nature of tumor angiogenesis. By studying many tumors of the same xenograft line, associations between MRI-derived parameters and parameters of the tumor microenvironment can be revealed. However, these MRI-derived parameters cannot be expected to be prognostic for the clinical outcome of cancer unless they show significant correlations with microenvironmental parameters across tumor lines. Interestingly, ADC showed significant inverse correlations with cell density, HF_Pim_, and IFP across the two melanoma lines included in this study, consistent with the hypothesis that tumor ADC may be an important prognostic factor in cancer.

The hypothesis that low ADC may indicate poor prognosis is also supported by the observation that low ADC was associated with pulmonary metastasis in A-07 tumors. On the other hand, the observation that R-18 tumors showed lower ADC values than the A-07 tumors and did not give rise to pulmonary metastases is apparently inconsistent with this hypothesis. Previous studies have revealed that the A-07 and R-18 xenograft lines show organ-specific metastatic patterns (i.e., A-07 tumors metastasize primarily to the lungs, whereas R-18 tumors preferentially develop lymph node metastases) [13]. The incidence of lymph node metastases was not scored in the present investigation. However, previous investigations have revealed that the development of lymph node metastases in R-18 tumors is associated with poor blood supply and high fractions of hypoxic tissue [35] and, furthermore, that hypoxia promotes lymph node metastasis in R-18 tumors primarily by up-regulating the expression of the urokinase-type plasminogen activator receptor [36].

## Conclusions

This study confirmed that low ADC is associated with high cell density in tumors and revealed that tumors with high cell densities may have high hypoxic fractions, high IFP, and elevated metastatic propensity. Low ADC was found to be associated with high HF_Pim_, high IFP, and metastasis, primarily because these parameters were all associated with high tumor cell density. Taken together, our observations suggest that low pretreatment ADC values may be associated with a hostile microenvironment in tumors. Consequently, the possibility that DW-MRI may provide biomarkers of microenvironment-associated tumor aggressiveness and treatment resistance merits clinical investigations.

## Abbreviations

ADC: Apparent diffusion coefficient; DW-MRI: Diffusion-weighted magnetic resonance imaging; ETL: Echo train length; HF_Pim_: Hypoxic fraction assessed by using pimonidazole as a hypoxia marker; IFP: Interstitial fluid pressure; TE: Echo time; TR: Repetition time.

## Competing interests

The authors declare that they have no competing interests.

## Authors’ contributions

TH was involved in conceiving the study, designing and performing experiments, analyzing and interpreting data, carrying out statistical analyses, and preparing the manuscript. CE was involved in designing experiments, interpreting data, and preparing the manuscript. KG was involved in performing experiments and analyzing and interpreting data. EKR was involved in conceiving the study, interpreting data, and preparing the manuscript. All authors read and approved the final manuscript.

## Pre-publication history

The pre-publication history for this paper can be accessed here:

http://www.biomedcentral.com/1471-2407/14/92/prepub
